# Immunosuppressive Exosomes: A New Approach for Treating Arthritis

**DOI:** 10.1155/2012/573528

**Published:** 2012-03-06

**Authors:** Chenjie Yang, Paul D. Robbins

**Affiliations:** Department of Microbiology and Molecular Genetics, University of Pittsburgh School of Medicine, Pittsburgh, PA 15219, USA

## Abstract

Rheumatoid arthritis (RA) is a chronic autoimmune disease and one of the leading causes of disability in the USA. Although certain biological therapies, including protein and antibodies targeting inflammatory factors such as the tumor necrosis factor, are effective in reducing symptoms of RA, these treatments do not reverse disease. Also, although novel gene therapy approaches have shown promise in preclinical and clinical studies to treat RA, it is still unclear whether gene therapy can be readily and safely applied to treat the large number of RA patients. Recently, nanosized, endocytic-derived membrane vesicles “exosomes” were demonstrated to function in cell-to-cell communication and to possess potent immunoregulatory properties. In particular, immunosuppressive DC-derived exosomes and blood plasma- or serum-derived exosomes have shown potent therapeutic effects in animal models of inflammatory and autoimmune disease including RA. This paper discusses the current knowledge on the production, efficacy, mechanism of action, and potential therapeutic use of immunosuppressive exosomes for arthritis therapy.

## 1. Introduction

Arthritis refers to joint inflammation resulting from an autoimmune disease, joint wear and tear, or bacterial/viral infection. Rheumatoid arthritis (RA) is a chronic, systemic autoimmune disorder in which endogenous synovial joints and other connective tissues are attacked by the immune cells. The pathological infiltration of inflammatory cells and synovial hyperplasia usually leads to the progressive destruction of articular cartilage and ankylosis of the joints. Although different types of treatment are available to alleviate symptoms and/or improve disease pathologies, no known therapy has been effective in reversing disease progression.

Conventional therapies for RA include nonsteroidal anti-inflammatory drugs, steroids, so-called “disease-modifying medications” (DMARDs), and surgery. These therapies are mostly palliative, can cause significant side effects, and offer no cure for the disease. In the early 1990s, biological therapies were demonstrated to alleviate symptoms of disease, but not to necessarily cure disease. Currently several different biologics, in particular inhibitors of tumor necrosis factor-*α* (TNF-*α*), are the leading drugs for treating RA. However, the need for constant infusion of drugs, either intravenously or subcutaneously, as well as the fact that not all patients response to anti-TNF therapy, necessitates the development of new RA therapies.

Gene therapy approaches also have been developed to treat arthritis and related joint disorders, demonstrated to be highly therapeutic in many animal models and safe in several clinical trials [1–3]. Gene transfer can be used to deliver genes encoding factors that inhibit proinflammatory cytokines (e.g., IL-1 receptor antagonist (IL-1Ra) for IL-1*β*  inhibition, TNF receptor for TNF-*α*  inhibition, and IL-18 neutralizer), Th2-polarizing and anti-inflammatory cytokines (e.g., IL-4, IL-10 and TGF-*β*), apoptosis-inducing factor (e.g., FasL), NF-*κ*B inhibitors or decoy oligodeoxynucleotides, or cartilage destruction inhibitor (e.g., Ribozymes and MMP-1 antisense construct), either locally to the inflamed joints or systemically [4–7]. The types of vectors for gene delivery include nonviral (e.g., plasmid DNA) and viral (e.g., retrovirus, adenovirus, adenoassociated virus, lentivirus and herpes simplex virus) vectors. The potential problems with *in vivo* gene transfer using viral vectors include possible toxicity and immunogenicity [8, 9]. Among these vectors, adenoassociated virus vector, which has limited immunogenicity and toxicity, has been used safely in several Phase I and II gene therapy trials for gene transfer of the TNF soluble receptor locally to joints.

*Ex vivo* gene transfer is an alternative gene delivery strategy where cells are genetically modified *in vitro* followed by local or systemic injection. The advantages of *ex vivo* gene transfer include better safety by controlling the type of cells transduced and reduced immunogenicity following injection. Synovial fibroblasts (SFs) or fibroblast-like synoviocytes (FLS) have been a major target for *ex vivo* gene transfer in RA therapy. Intra-articular injection of virally transduced SFs expressing IL-1Ra was therapeutic in animal models of arthritis [10–12] and a clinical study using IL-1Ra-transduced autologous RASFs for injection into knuckle joints of RA patients showed evidence of therapeutic effects without any adverse events [13]. Still, RASFs have the problems of low transduction efficiency and low proliferation rate which hamper their application on a large scale [14]. In addition to RASFs, dendritic cells (DCs, discussed below) as well as antigen-specific T cells [15] have also been used as vehicles to deliver immunosuppressive cytokines for the treatment of collagen-induced arthritis (CIA) in mouse models.

 DCs are antigen-presenting cells (APCs) derived from CD34^+^ stem cells that can regulate immune reactivity. Although DC were initially considered as instigators of immune responses, including organ graft rejection and autoimmune disorders, more recent data have implicated DC in the induction and even maintenance of tolerance to allo- or autoantigens in experimental models. The ability of DC to either stimulate or suppress immune responses is mediated by various factors; the most important being their stage of differentiation/maturation/activation and their hematopoietic lineage affiliation. Immature DCs, characterized by low levels of MHC and costimulatory molecules (e.g., CD80, CD86, CD40, ICAM-1, and ICOSL), are able to suppress antigen-specific T-cell responses. These “tolerogenic” DCs produce reduced levels of type 1 cytokines (e.g., IL-12 family members) and increased level of immunosuppressive cytokines (e.g., IL-10, TGF-*β*, and VEGF). They can also express high levels of the tryptophan catabolizing enzyme indoleamine 2,3-dioxygenase (IDO), which degrades free tryptophan and “starves” responder T cells of the essential amino acid, resulting in increased T-cell apoptosis [16]. The mechanisms through which tolerogenic DCs exert regulatory functions can include the induction of antigen-specific T-cell anergy or deletion [17]; induction of regulatory T cells [18–20]; polarization of T cells away from a Th1 or Th17-type response and toward a Th2-type response [21]. The immunosuppressive/tolerogenic properties of DCs can be enhanced and stabilized by genetic modification. Genetically modified DCs have been shown to successfully ameliorate symptoms and control disease progression in animal autoimmune disease models including RA and type 1 diabetes [22–26]. In fact, DCs transduced with viral vectors expressing immunosuppressive agents were found to be more effective in treating murine CIA than similarly modified fibroblasts or T cells [22, 27].

**Figure 1:**
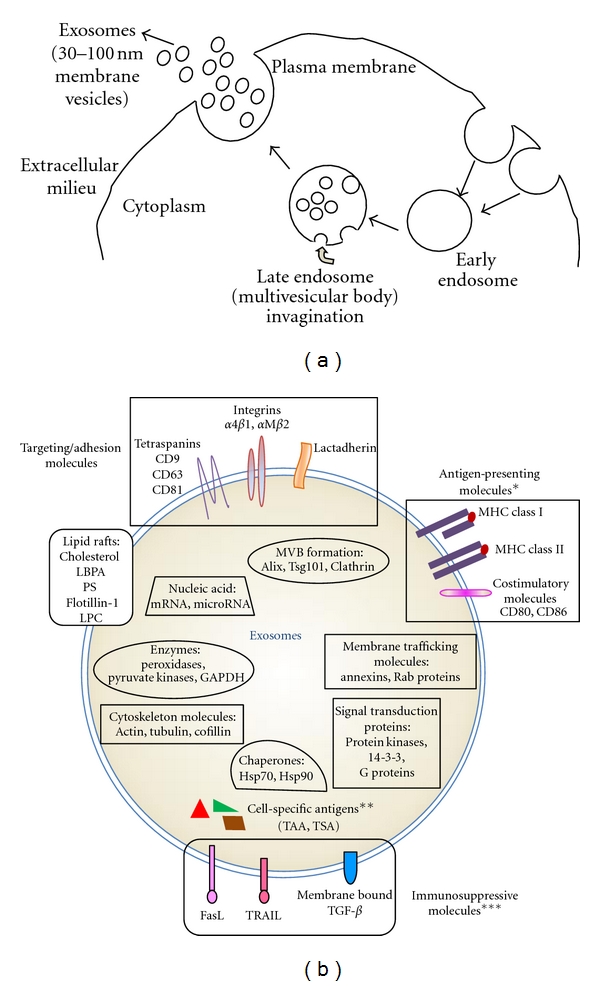
Exosome biogenesis and molecular composition. (a) Exosomes are small membrane vesicles formed by invagination of the multivesicular bodies (MVBs) in the late endocytic compartment. They are released upon the fusion of MVBs with the plasma membrane. (b) Exosomes are typically enriched in certain molecules including targeting/adhesion molecules, membrane trafficking molecules, cytoskeleton molecules, proteins involved in MVB formation, chaperones, cytoplasmic enzymes, signal transduction proteins, and functional mRNA and microRNA populations. *APC-derived exosomes contain antigen-presenting molecules including MHC class I, MHC class II, and co-stimulatory molecules. **Exosomes also contain cell-specific antigens (e.g., tumor antigens in tumor-derived exosomes). ***Immunosuppressive molecules such as FasL, TRAIL, or TGF-*β*  are present on certain APC or tumor-derived exosomes.

**Figure 2:**
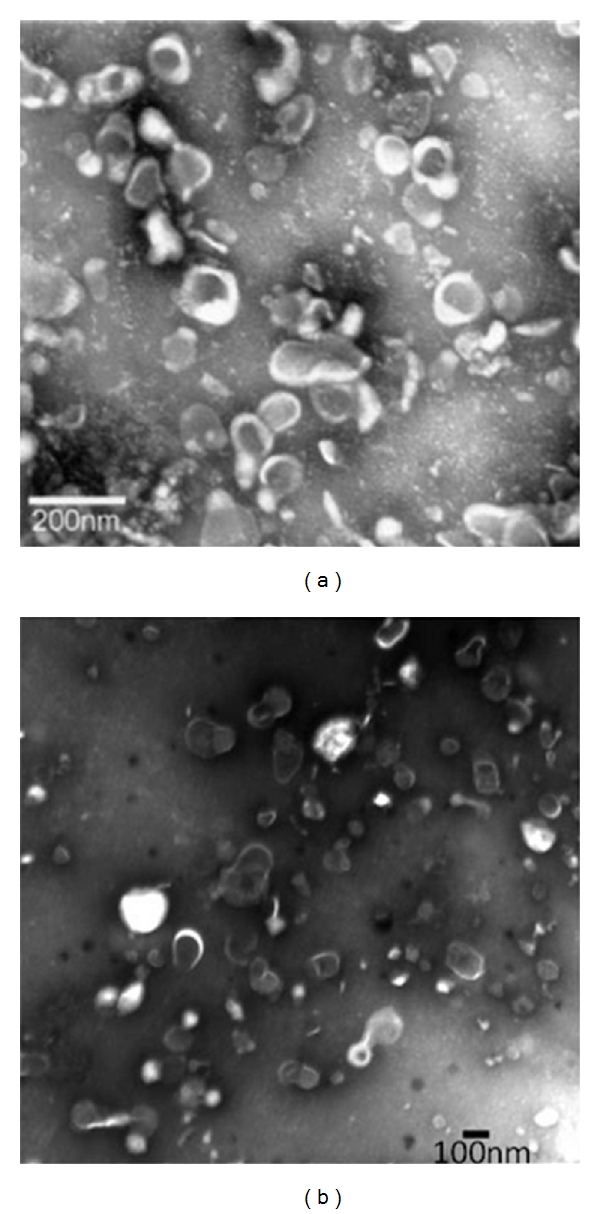
Transmission electronic micrograph of (a) exosomes isolated from murine BMDC culture [36].* Copyright 2005. The American Association of Immunologists, Inc.* and (b) exosomes isolated from murine blood plasma [37]. *Copyright 2007. The American Association of Immunologists, Inc*.

More recently, exosomes derived from immunosuppressive DCs have been found to confer potent and lasting immunosuppressive effects, similar to their parental DC. Exosomes are a type of secreted membrane vesicles produced by most cell types. They are characterized by a size of 30–100 nm in diameter and an endocytic origin, formed by the reverse budding of the multivesicular bodies and released upon their fusion with the plasma membrane [28–30] (Figure 1(a)). Their protein content largely reflects that of the parental cells and is enriched in certain molecules including adhesion molecules, membrane trafficking molecules, cytoskeleton molecules, heat-shock proteins, cytoplasmic enzymes, signal transduction proteins, and cell-specific antigens [28, 31, 32]. APC-derived exosomes are enriched in MHC classes I and II as well as co-stimulatory molecules. Exosomes also contain functional mRNA and microRNAs molecules [33–35] (Figure 1(b)). Most hematopoietic cells, including DCs, produce copious amount of exosomes. Certain types of exosomes have been shown to confer immunosuppressive effects in different disease models including RA. Thus it is likely that exosomes represent a novel effective and safe therapeutic approach for treating arthritis. Indeed, exosomes derived from immunosuppressive DCs and from peripheral blood (Figure 2) have shown the ability to suppress inflammation.

## 2. Immunosuppressive DC-Derived Exosomes for Arthritis Treatment

### 2.1. DC/IL-10 Exosomes

DCs transduced with adenovirus expressing the IL-10 gene (Ad.IL-10) or treated with recombinant murine IL-10 (rmIL-10) were demonstrated to be anti-inflammatory and suppress mouse CIA [36, 38]. Interestingly, exosomes secreted by those DCs were also found immunosuppressive. Exosomes derived from Ad.IL-10 DCs were capable of decreasing T-cell proliferation in a mixed lymphocytes reaction. In mice immunized with keyhole limpet hemocyanin (KLH) antigen, local injection of DCs (ad.vIL-10 or rmIL-10 treated) or their exosomes both resulted in significant suppression of KLH-induced delayed-type hypersensitivity (DTH) response. Moreover, a single dose of these exosomes systemically delivered after the onset of CIA effectively ameliorated disease progression, in contrast to the ineffectiveness of direct injection of rmIL-10 [36]. Similar efficacy was observed using DC/IL-10 exosomes to suppress DTH response and CIA compared with DC/IL-10 cell treatment, making DC exosomes an even attractive therapy than DCs.

Although not fully understood yet, the suppressive effect of DC/IL-10 exosomes is not simply due to the delivery of the suppressive cytokine IL-10. Instead, the therapeutic effect of exosomes requires the integrity of the exosome membrane, as repeated freeze and thaw cycles that disrupt the exosome structure abrogated the effect. The effect is also MHC class II dependent since exosomes deficient in MHC class II did not suppress DTH [36]. In addition, the presence of B7-1/2 (CD80 and CD86) on IL-10-treated BMDC-derived exosomes is required for their suppressive effects [39]. Thus it is possible that IL-10 treatment results in DC-derived exosomes with a different composition that makes the vesicles more immunosuppressive.

### 2.2. DC/IL-4 Exosomes

We and others have demonstrated that DCs genetically engineered to express the Th2 cytokine IL-4 were an effective treatment for murine CIA [22, 23]. Specifically, we found that a single i.v. injection of immature BMDCs infected with adenoviral vector expressing IL-4 into mice with established CIA achieved almost complete suppression of the disease lasting at least 4 wk posttreatment. The therapeutic effect of the IL-4 expressing DC (DC/IL-4) was significantly better than repeated injection of recombinant IL-4 or direct injection of adenoviral IL-4 [23]. DCs retrovirally transduced with the IL-4 gene also reduced the incidence and severity of CIA after a single i.p. injection [22]. In both studies, DC/IL-4 reduced the disease-associated humoral responses and conditioned splenic cells towards a Th2-polarized response upon antigen stimulation.

Similar to the significant immunosuppressive effects of DC/IL-4, exosomes derived from those DCs were shown to reduce the severity and the incidence of established CIA when delivered systemically (i.v.), and suppressed DTH response when injected locally [40]. Suppression of the DTH response is MHC restricted in that only syngeneic DC exosomes, but not allogeneic exosomes, were effective in conferring immunosuppression. Furthermore, systemically injected DC/IL-4 exosomes were found to migrate to spleen and liver and interact with CD11c+ DCs and F4/80+ macrophages. Adoptive transfer of CD11c+ or CD3+ splenic cells isolated from antigen-immunized mice that have been systemically treated with exosomes into the footpad of recipient mice significantly reduced footpad swelling in the DTH model, suggesting that exosomes from DC/IL-4 can directly or indirectly modify the function of endogenous APCs and T cells, either by inducing a regulatory subset and/or depleting antigen-reactive Th1 cells [40].

### 2.3. DC/Death Ligand Exosomes

Selective inducing apoptosis of antigen-specific T cells by APCs genetically modified to express death ligand (e.g., FasL and TRAIL) is an alternative way to downregulate antigen-specific T-cell responses. DCs genetically modified to express FasL can induce donor-specific T-cell hyporesponsiveness to alloantigen and facilitate allograft survival [41]. FasL-expressing DCs are also able to suppress collagen-reactive T cells and inhibit the progression of murine CIA after systemic injection [26]. Exosomes derived from FasL-expressing DCs showed an anti-inflammatory effect in a murine DTH model upon local administration [42]. The therapeutic effect was abolished when *lpr* (Fas-deficient) mice were used as recipients or when exosomes were derived from the DCs of *gld* (FasL-deficient) mice. However, the immunosuppressive effect of FasL-deficient DC exosomes could be restored by gene transfer of FasL to DCs. The ability of DC/FasL exosomes to suppress DTH response was also antigen specific as optimal suppressive effects were achieved when the DCs were prepulsed with the same antigen used for mice immunization. Using DC and DC-derived exosomes from different knockout mice, the suppressive effect was shown to be MHC class II dependent, but MHC class I independent. Systemic injection of DC/FasL exosomes was also effective in treating established murine CIA [42].

It was shown that infiltrating T cells present in the synovial fluid of RA patients were more susceptible to apoptosis induced by APO2L/TRAIL, a TNF superfamily member capable of inducing cell apoptosis. Bioactive APO2L/TRAIL associated with exosomes was detected in the synovial fluid of RA patients compared with synovial fluid of traumatic arthritis patients [43]. Interestingly, bioactive APO2L/TRAIL conjugated to the membrane of liposomes, artificial lipid vesicles resembling exosomes, was demonstrated to substantially reduce inflammation after intra-articular injection in a rabbit model of RA, more effectively than soluble, unconjugated APO2L/TRAIL. The increased bioactivity is possibly due to the enhanced receptor cross-linking as a result of increased local concentration of the protein upon liposome delivery [44].

### 2.4. DC/IDO Exosomes

Immunoregulatory DCs expressing the tryptophan catabolic enzyme IDO can inhibit T-cell activation and suppress T-cell responses to auto- and alloantigens by tryptophan starvation and/or production of toxic metabolites [45, 46]. The immunosuppressive potency of DCs genetically modified to express IDO and their exosomes was also investigated in RA models. BMDCs adenovirally transduced to express IDO and the resulting DC/IDO exosomes both showed anti-inflammatory effect in murine DTH and CIA models. In addition, transduction of DCs with the IDO inducer CTLA4-Ig resulted in induction of IDO and the derived exosomes were also able to reduce inflammation [47]. The suppressive effect of DC/CTLA4-Ig exosomes was reduced when DCs were pre-treated with the competitive IDO inhibitor 1-MT or excessive L-tryptophan, suggesting that the effect was dependent on the IDO activity in DCs and IDO-mediated tryptophan deprivation. Similar to exosomes derived from DC/IL-10, the immunosuppressive effect of DC/IDO exosomes was partially dependent on B7-1/2 molecules, as exosomes of DCs isolated from B7-1/2 knockout mice had an attenuated anti-inflammatory effect when transduced with IDO [47].

### 2.5. Mechanism(s) of Exosome Function

Although the exact functional mechanism of immunosuppressive DC-derived exosomes remains to be determined, exosomes are believed to function more than vehicles that simply deliver immunosuppressive factors derived from their parental DCs. This is evidenced by the facts that most of the suppressive effects observed have antigen specificity and are dependent on the presence of certain molecules on exosomes as well as in recipient animals, in particular MHC class II molecules and B7-1/2. Furthermore, similar to direct gene transfer or DC cell therapy [38, 48–51], distal therapeutic effects (contralateral effects) were observed when exosomes were delivered locally. However, trafficking analysis suggested that there is only limited cross-trafficking of exosomes to the contralateral lymph node [42]. Therefore, it is likely that immunosuppressive DC exosomes are able to modify the behavior of endogenous immune cells, such as APCs, which then are responsible for conferring a systemic suppressive/anti-inflammatory effect. The interactions between exosomes and APCs could be at the membrane level or, in some cases, involve the internalization of these vesicles where vesicle-contained proteins and RNAs could be functionally transferred.

## 3. Immunosuppressive Exosomes in Body Fluids

In addition to exosomes derived from immunosuppressive DCs, other sources of suppressive exosomes may also have the potential to treat inflammatory arthritis diseases. Many types of tissue- or body-fluid-derived exosomes have been found to be immunoregulatory or tolerogenic. For instance, placenta-derived exosomes and exosomes isolated from the maternal peripheral circulation are able to induce T-cell signaling defects, possibly attenuating immune responses against the fetus [52, 53]. Exosomes isolated from serum shortly after antigen feeding are able to induce antigen-specific tolerance in naïve recipient animals [54]. In addition, exosomes isolated from the bronchoalveolar fluid of mice respiratory exposed to pollen allergen can prevent antigen-specific allergic reaction [55]. Also, exosomes isolated from human breast milk and colostrum can increase the number of T regulatory cells and inhibit effector T-cell activation *in vitro *[56]. Interestingly, exosomes derived from certain body fluids, in particular conditioned blood plasma or serum, have been found to reduce arthritic inflammation.

### 3.1. Plasma-Derived Exosomes

We have demonstrated that exosome-like vesicles can be isolated from the blood plasma of both naïve mice and antigen-immunized mice, with certain surface protein markers including MHC class I, MHC class II, CD11b, CD71, FasL, and CD86. Plasma-derived exosomes isolated from mice immunized with KLH antigen showed potent suppressive effect on the KLH-induced DTH response after local administration [37]. The effect was antigen specific since plasma-derived exosomes isolated from mice immunized with an irrelevant antigen did not induce effective suppression. MHC class II+ exosomes were responsible for conferring the suppressive effect as depletion of MHC class II+ exosomes from plasma-derived exosomes abrogated this effect. The anti-inflammatory effect also required Fas/FasL signaling. Additionally, the effect was time dependent since the optimal immunosuppressive activity was obtained with exosomes isolated 14 days after immunization. This result suggests the presence of exosomes with antigen-specific immunosuppressive activity in the circulation of individuals that are hyperreactive to certain antigens. It also suggests the possibility of utilizing autologous plasma-derived exosomes therapeutically for the suppression of antigen-specific inflammation.

### 3.2. Clinical Studies with Serum-Derived, Anti-Inflammatory Exosomes

An effective method for stimulating *de novo* production of anti-inflammatory cytokines in blood was developed by incubating whole blood with CrSO4-treated glass beads for a short period of time [57]. Such treatment resulted in the robust induction of IL-1Ra and an increase in the levels of IL-4 and IL-10 as well as certain growth factors such as insulin-like growth factor-1 (IGF-1). This approach for the preparation of autologous conditioned serum (ACS) with enhanced anti-inflammatory cytokines has been used for the clinical treatment of patients with RA, osteoarthritis (OA), and spinal disorders, with efficacy and safety both observed [58, 59]. Exosomes with anti-inflammatory properties were isolated from ACS and have been tested clinically for the treatment of RA. Intra-articular injection of these exosomes appears to be therapeutic for RA patients who do not respond well to conventional therapy, with reduced pain in multiple joints and decreased inflammatory markers in the blood [60]. The therapeutic use of these ACS-derived exosomes also appears to be safe, supporting the development of similar exosome treatments for other inflammatory and autoimmune diseases. The immunosuppressive exosomes shown to be effective in treating murine and human RA are summarized in Table 1.

## 4. Pathogenic Exosomes

We have discussed the potential usage of immunosuppressive exosomes for arthritis treatment. However, additional studies have also suggested that exosomes of certain sources could contribute to disease progression. For example, exosomes produced by the synovial fibroblasts obtained from RA patients were found to contain a membrane form of TNF-*α*, which could play a role in tissue destruction and autoimmune inflammation. These TNF-*α*  positive exosomes rendered activated T cells resistant to apoptosis, favoring the pathogenesis of RA [61]. In addition, citrullinated proteins, known to be autoantigens in RA, were detected in exosomes purified from the synovial fluids of RA patients [62]. Similarly, the autoantigen nuclear protein DEK, which contributes directly to joint inflammation in juvenile arthritis (JA), is secreted in both a free form and an exosome-associated form in the synovial fluids of JA patients [63, 64]. Exosomes containing annexins, which promote pathological mineral formation and articular chondrocytes destruction, were found more in the articular cartilage in OA patients [65]. These observations demonstrate the presence of disease-contributing exosomes, which could be useful inflammation markers of arthritis diseases. In theory, selective elimination of these exosomes would be beneficial to arthritis therapy.

## 5. Conclusion

Immature DCs with enhanced immunosuppressive/tolerogenic properties can be produced by genetic modification or by exposure to cytokines or cytokine inhibitors. Exosomes derived from immunosuppressive DCs have shown therapeutic effects comparable to or better than their parental DCs in treating animal DTH and CIA and thus have the potential to be used clinically for RA treatment. While DCs manipulated *ex vivo* still have the risk of maturation in inflammatory environment, DC-derived exosomes are more stable following isolation and thus are safer than autologous cells for *in vivo* administration. The unique biological composition of exosomes also gives them a half-life that appears longer than many cell types after injection. However, the paucity of clinical trials using immunosuppressive DC exosomes for arthritis treatment still prevents a comprehensive evaluation of their effects on human patients.

The fact that exosomes isolated directly from ACS appears to improve disease in RA patients strongly supports the further clinical development of immunosuppressive exosomes. However, it is important to note that while the clinical results are encouraging in terms of feasibility, safety, and efficacy, the blood plasma- or serum-derived exosomes have heterogeneous cellular origins and poorly defined composition. Further investigation is needed to determine the functional components of these therapeutic exosomes. Taken together, there is considerable evidence supporting the ability of immunosuppressive exosomes to help control the overreactive immune system. Compared with gene and cell therapies, exosome-based therapy could provide a new and safe therapeutic approach for arthritis.

## Figures and Tables

**Table 1:** Immunosuppressive exosomes for the treatment of arthritis.

Exosome source	Cell modification/treatment	Model	Application and effect	Reference
BMDC	BMDCs transduced with adenoviral IL-10 or treated with rmIL-10	Mouse	Footpad injection suppressed DTH response; systemic delivery (i.v.) ameliorated CIA progression. The effect requires exosome integrity and MHC class II and B7-1/2 molecules.	[36, 39]

BMDC	BMDCs transduced with adenoviral IL-4 or retroviral IL-4	Mouse	Systemic injection (i.v.) reduced the incidence and severity of established CIA; local injection suppressed DTH response. Exosomes interacted with DCs and macrophages in spleen and liver and were able to modify the function of endogenous APCs and T cells.	[40]

BMDC	BMDCs transduced with adenoviral FasL	Mouse	Local administration suppressed DTH response. The effect was dependent on Fas-FasL interaction and MHC class II molecules. Systemic injection was also effective in treating established CIA.	[42]

BMDC	BMDCs transduced with adenoviral IDO or CTLA4-Ig	Mouse	DC/IDO exosomes were anti-inflammatory in both DTH and CIA models. DC/CTLA4-Ig exosomes reduced DTH response. The effect was dependent on the IDO activity in DCs and partially dependent on B7-1/2 molecules.	[47]

Blood plasma	Exosomes were isolated from the plasma of antigen-immunized mice	Mouse	Local administration of plasma-derived exosomes suppressed DTH response in an antigen-specific manner. The effect was dependent on MHC class II+ exosomes.	[37]

Serum	Exosomes were isolated from physicochemically conditioned autologous patient serum	Human clinical trial	ACS exosomes exhibited anti-inflammatory properties. Local injection of these exosomes was safe and beneficial to RA patients that do not respond to conventional therapy. Reduced joint pain and decreased blood inflammatory markers were observed.	[58–60]
